# Literary Notes

**Published:** 1904-02

**Authors:** 


### LITERARY NOTES.

The Cincinnati *Lancet-Clinic* enters upon the new year with an entire change in its editorial management. Dr. J. C. Culbertson, who has so long and ably conducted the journal, retires and Dr. Mark A. Brown assumes editorial control. Dr. Frank B. Cross becomes the business manager, and a long list of names, many of them well known in the annals of medicine, are announced as a staff of associate editors.

Dr. Culbertson is entitled to a rest after thirty years or more of continuous editorial work. We wish him prosperity and continued good health, while for the *Lancet-Clinic* we offer our best congratulations for a prolongation of its successful career under the new management.

---

The *Medical Fortnightly* presents itself in a new dress in its first issue for 1904, dated January 11. Its appearance is very much improved and is in every way a credit to those concerned in its editorial and business management.

THE *Medical Recorder* is the name of a new journal published at Shreveport, La. It is edited by Dr. Oscar Dowling as principal, and Drs. Louis Abramson, of Shreveport, and R. H. T. Mann, of Texarkano, Ark., as associates. The first number bears date January, 1904, and contains 57 standard octavo pages. It also contains much material of medical interest and several obituary notices with half-tone plates of deceased physicians. It is especially intended to reach the states of Louisiana, Texas, and Arkansas and, no doubt, will be greatly appreciated, not only by physicians in those states, but throughout the country.

